# Compressive holographic sensing simplifies quantitative phase imaging

**DOI:** 10.1038/s41377-023-01145-y

**Published:** 2023-05-17

**Authors:** Jiawei Sun, Juergen W. Czarske

**Affiliations:** 1 Competence Center for Biomedical Computational Laser Systems (BIOLAS), TU Dresden, Dresden, Germany; 2 Laboratory of Measurement and Sensor System Technique (MST), TU Dresden, Helmholtzstrasse 18, 01069 Dresden, Germany; 3 Cluster of Excellence Physics of Life, TU Dresden, Dresden, Germany; 4 Institute of Applied Physics, TU Dresden, Dresden, Germany

**Keywords:** Optics and photonics, Electronics, photonics and device physics

## Abstract

Quantitative phase imaging (QPI) has emerged as method for investigating biological specimen and technical objects. However, conventional methods often suffer from shortcomings in image quality, such as the twin image artifact. A novel computational framework for QPI is presented with high quality inline holographic imaging from a single intensity image. This paradigm shift is promising for advanced QPI of cells and tissues.

## Abstract (web summary)

Quantitative phase imaging (QPI) has emerged as method especially for investigating biological specimen. However, conventional methods often suffer from shortcomings in image quality, such as the twin image artifact of inline holography. The presented compact 3D sensing system utilizes a novel computational framework for QPI from single intensity images. The reported system offers accurate and efficient 3D profiling, overcoming the limitations of conventional inline holography approaches. This paradigm shift is promising for biomedical imaging, and material analysis.

## Graphical abstract

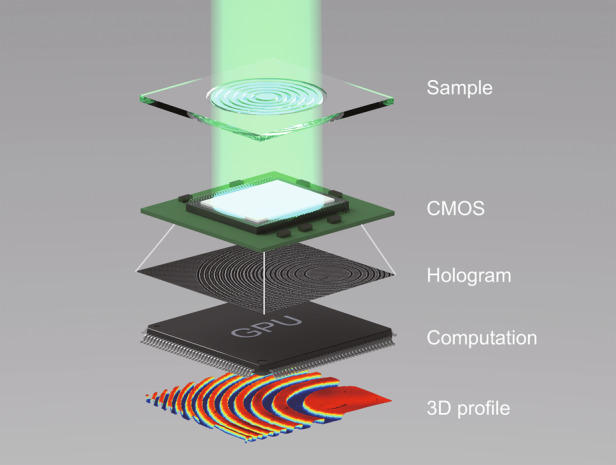

Digital holography has become a powerful non-destructive imaging technique in engineering, and biomedicine^1–3^. Unlike traditional imaging techniques that only capture intensity information, holography provides both amplitude and phase information of the reflected or transmitted light field from an object. This feature enables the reconstruction of high-resolution, three-dimensional images that accurately represent the object’s shape and structure with nanoscale precision. Nevertheless, a twin image is generated in the reconstruction process due to the complex conjugate nature of the digital holography^4^. The twin image is a significant problem in digital holography as it overlaps with and obscures the measured object, leading to reduced image quality and contrast. Such twin image problem can be physically suppressed by off-axis holography^5^, where the twin images are separated in Fourier space by introducing a small angle in between the reference beam and the object beam. Off-axis digital holography allows the twin image to be filtered out numerically, but this approach can result in sacrificing the space bandwidth product of the system. To overcome the twin image problem, another effective approach is to take multiple measurements, introducing variations in the imaging distance^6^, illumination wavelength^7^, probe position^8^, modulation pattern^9^, and illumination angle^10^. However, the high imaging performance of these approaches often comes at the expense of a lower temporal resolution and sophisticated complex optical system. There is growing interest in developing non-interferometric, single-shot digital holography techniques towards compact systems for quantitative phase imaging (QPI).

Now, writing in an issue of *Light: Advanced Manufacturing*, Yunhui Gao and Liangcai Cao from Tsinghua University report a novel computational framework that achieves quantitative phase reconstruction from a single inline hologram^11^.

Retrieving the optical phase and amplitude from a diffraction pattern alone has been a long-standing problem. Previously, researchers proposed exploiting the physical knowledge of the wavefield as a simple yet efficient constraint to suppress the twin image^12^. Later on, with the advent of compressive sensing and deep learning, more advanced image priors based on the regularization techniques have also been explored in the context of phase retrieval^13–15^.

However, both of these methods have been studied separately and feature their unique advantages. In this work, Gao et al. proposed an inverse problem approach that encapsulates both physical constraints and sparsity priors within a unified framework. Specifically, the well-known absorption and support constraints, which enforce the wavefield to satisfy the underlying physics, are introduced. Meanwhile, a total variation function is used as a sparsity-promoting regularizer to characterize the piecewise smoothness of the samples. Combining the two leads to a constrained complex total variation (CCTV) model, which can be solved by an accelerated proximal gradient algorithm with an efficient denoiser.

The lensless hologram is recorded directly by an imaging sensor (CMOS) without the need for lenses, enabling an ultra-compact system. Powered by the novel computational framework reported in ref. ^11^, this system is capable of calculating accurate 3D profiles with nanoscale resolution. Based on a simple lensless system demonstrated in Fig. 1, the authors experimentally demonstrated the QPI capability of the CCTV model on various samples, ranging from biological tissue slides such as muscle tissue to fabricated transparent phase plates. The imaging accuracy has also been quantified by imaging standard amplitude and phase test targets, and the results show good consistency with the ground-truth data. Comparative experiments show that using sparsity regularization can effectively suppress the twin image, while the physical constraints can help further accelerate the algorithm and enhance overall fidelity. Another important insight gained through this work is the convergence behavior of the compressive phase retrieval algorithm. Based on the geometrical properties of the inverse problem, the authors established a general convergence theory that ensures stable convergence of the algorithm with pre-specified parameters. This implies that the proposed algorithm is essentially different from most heuristic phase retrieval algorithms with theoretically tractable algorithmic behaviors. To facilitate further studies, the authors have published open source code and experimental data along with the paper^16^. They also elaborate on the parameter selection rules, providing guideline for fast and easy implementation by practitioners of various backgrounds.

**Fig. 1:**
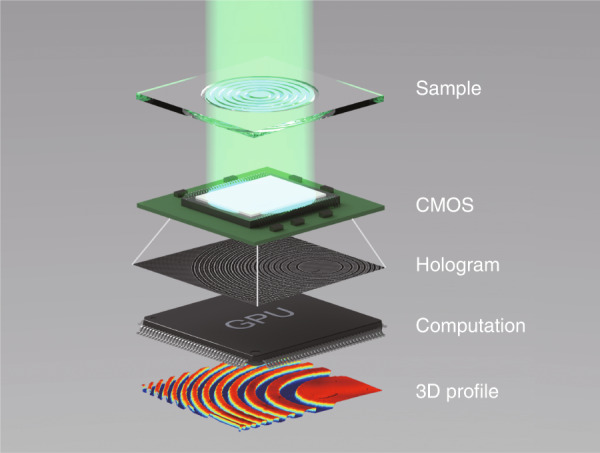
Working principle of the compact 3D profile sensing system

The compressive phase retrieval framework proposed in this work has the potential to inspire both theoretical and empirical studies in the future. Despite the iterative reconstruction process, the algorithm allows highly parallel computation and can be accelerated by orders of magnitude with the help of graphical processing units. The computational approach presented in this paper can be readily applied to a wide range of fields, including coherent diffraction imaging^17^, optical diffraction tomography^18,19^, holographic endoscopy^6,20^, holographic optical manipulation^21,22^, and biomaterial characterization^23^.
